# Comparison of individuals with low versus high consumption of home-prepared food in a group with universally high dietary quality: a cross-sectional analysis of the UK National Diet & Nutrition Survey (2008–2016)

**DOI:** 10.1186/s12966-019-0768-7

**Published:** 2019-01-17

**Authors:** Chloe Clifford Astbury, Tarra L. Penney, Jean Adams

**Affiliations:** MRC Epidemiology Unit & Centre for Diet and Activity Research (CEDAR), University of Cambridge, Box 285, Institute of Metabolic Science, Cambridge Biomedical Campus, Cambridge, CB2 0QQ UK

**Keywords:** Home-prepared food, Food practices, Cooking, Cooking skills, Food skills, DASH, Diet quality

## Abstract

**Background:**

Despite inconclusive evidence, the idea that a lack of home food preparation and skills is a limiting factor in achieving a healthy diet is widespread. Cooking skills interventions proliferate, and several countries now mention cooking in their dietary guidelines. The aim of this study was to determine whether substantial consumption of home-prepared food is necessary for high dietary quality by exploring whether individuals can eat healthily while eating little home-prepared food. The diets of these individuals were characterised, and socio-demographic characteristics and prevalence of obesity were also explored.

**Methods:**

Cross-sectional analysis of UK dietary survey data with objectively measured height and weight and a 4-day food diary for each participant was conducted. A subsample (*N* = 1063, aged ≥19 years) with a high dietary quality (determined using a score derived from the Dietary Approaches to Stopping Hypertension (DASH) diet) was analysed. Within this, participants were grouped as either high or low home preparation based on the proportion of energy derived from home-prepared food. Regression models were used to determine whether and how those in the high and low home preparation groups differed in terms of socio-demographic characteristics, DASH score, energy intake, prevalence of obesity, and dietary composition.

**Results:**

The low home preparation group included 442 participants, while 621 participants were in the high home preparation group. The low home preparation group were more likely to be older and white, and less likely to have a degree level education. After adjustment for socio-demographic characteristics, there were no differences in DASH score, energy intake or obesity prevalence between the groups. After adjustment, the low home preparation group consumed more fruit (30.8 additional g/day, 95% CI 5.5–56.1), more low-fat dairy foods (24.6 additional g/day, 95% CI 1.7–47.5) and less red meat (10.4 fewer g/day, 95% CI 4.3–16.6), but also more sugar (11.6 additional g/day, 95% CI 7.5–15.6) and sodium (107.8 additional mg/day, 95% CI 13.8–201.8).

**Conclusion:**

Home food preparation should not be presented as a prerequisite to a high quality diet. The public health community should recognise the existence of a set of food practices which allows individuals to achieve a healthy diet with little contribution from home-prepared food, and make space for it in the design of their policies and interventions.

## Background

Given the significant contribution of diet to the ever-growing burden of chronic disease, [1] and the potential role a healthy diet could have in preventing overweight and obesity, heart disease, cancer and diabetes, [2–6] improving dietary quality at the population level emerges as one of the most urgent tasks for public health.

The rise in diet-related disease arrives in tandem with an increase in the consumption of food prepared outside the home [7, 8]. A decrease in time spent cooking at home has also been observed in most high-income countries, [9, 10] though there is evidence from the United States that the latter trend has been reversed since the early 2000s [11]. The consumption of food prepared outside the home is known to be associated with harmful dietary behaviours and negative health outcomes [12–14]. In contrast, there is some evidence, albeit less consistent, to suggest that higher frequency of both cooking [15–19] and eating home-prepared meals [20] is associated with better dietary intake and improved health outcomes.

There is significant interest from academics and policymakers in understanding the modifiable determinants of both these behaviours, and seeking ways of promoting the consumption of home-prepared food [21–24]. Countries such as Brazil [25] and Japan [26] have included cooking and food and cooking skills in their dietary guidelines, while Canada’s revised food guide will emphasise building food-related ‘skills and knowledge’ in its population as a means of improving dietary quality [27].

Further downstream, cooking and food classes and workshops are a popular public health intervention, and a wide variety of models run by governmental bodies, charities and social enterprises proliferate in countries such as the UK, the US, and Australia [28–30]. These interventions target a number of different demographics, and include a variety of components, including cooking workshops but also supermarket visits, nutrition classes, tasting sessions and work in kitchen gardens, deployed singly or in combination.

However, evidence of the effectiveness of these interventions is equivocal: systematic reviews conclude that evidence of significant and lasting change in either dietary behaviours or related health outcomes is limited [28–30]. Authors suggest this may in part be due to limitations in the design of both the interventions and their evaluations, but nevertheless existing evidence suggests that getting people to cook more or differently is difficult. With this in mind, it seems reasonable to pose the question: is substantial consumption of home-prepared food necessary for a healthy diet?

Promoting home cooking as a dietary public health intervention is based on the hypothesis that people who cook more have healthier diets and better health outcomes, an idea supported by some, [15–20] though admittedly not all, [31–34] of the evidence. However, preparing and eating food at home is complex, and the nutritional content of home-prepared meals can be highly variable, as can the nutritional content of meals prepared outside the home. One study showed that popular ready meals came closer to meeting dietary guidelines than homemade equivalents made using recipes from television chefs (though neither met the guidelines under study) [32]. Another study reported no significant difference between the healthfulness of ready meals and meals made at home using recipes from popular online sources and cookery books [35]. In a longitudinal, multi-ethnic study of midlife women, women who spent more time on meal preparation were more likely to develop metabolic syndrome, leading the authors to conclude that public health interventions should emphasise healthfulness of cooking as opposed to just cooking frequency [31].

Though there is some evidence that eating more home-prepared food is associated with better dietary quality, [20] the association between eating home-prepared food and dietary quality may be heterogeneous depending on what exactly is eaten.

An earlier (recently replaced) version of France’s *Guides alimentaires du programme national nutrition-santé* (national dietary guidelines) [36] proposed recommendations for different types of eaters, including for individuals who ‘do not cook’. Suggestions included bread and cereals, salad, fruit, milk and cheese. This seems to reflect a belief that it is possible to achieve a high quality diet while eating food that requires little or no home preparation. To the best of our knowledge, this hypothesis has not been quantitatively examined.

The aims of this study were (1) to determine whether substantial consumption of home-prepared food is for high dietary quality by exploring whether individuals can achieve a relatively high quality of diet while obtaining a relatively low proportion of their energy from home-prepared food; and (2) to characterise the diets of these individuals, if found, relative to their counterparts who also achieved a high quality diet while consuming a relatively large proportion of energy from home-prepared food. Individual-level socioeconomic and demographic characteristics of the two groups were also compared, as well as prevalence of overweight or obesity.

## Methods

This study represents a cross-sectional analysis of dietary surveillance data from the UK National Diet and Nutrition Survey (NDNS) 2008–16 (May 2018 release) [37]. It is reported according to the STROBE-nut recommendations [38].

### Data source

NDNS is an annual cross-sectional survey which collects information on food consumption and nutritional and health status of free-living individuals in the UK. Sampling, recruitment and data collection are carried out in a consistent manner, allowing data from different survey years to be combined for cross-sectional analysis.

A detailed account of the NDNS recruitment and sampling protocol has been published elsewhere [39–41]. In short, private addresses were randomly selected from postcode sectors across the UK. Within each household, a maximum of one adult and one child were randomly selected for inclusion in the study. These individuals were asked to complete a four-day food and drink diary, and to participate in an interview concerning more general dietary habits, socio-demographic status, lifestyle and physical activity, and receive a nurse visit which included measurement of height and weight.

NDNS was approved by the Oxfordshire Research Ethics Committee and written informed consent was obtained from all participants.

### Inclusion criteria

Individuals aged ≥19 years at the time of participation, who completed three or four days of the food diary, were included in the analyses. In order to compare those who achieved a relatively high dietary quality with and without a relatively high proportion of energy from home-cooked foods, only a sub-sample of the NDNS sample (the analytic sample) was included in this analysis: those in the top tertiles of both proportion of energy from home-prepared food and dietary quality (hereafter the high home preparation group), and those in the top tertile of dietary quality and the bottom tertile of energy from home-prepared food (the low home preparation group). This resulted in an analytic sample with universally high dietary quality, allowing inter-group differences to be associated with consumption of home-prepared food as opposed to dietary quality.

### Dietary assessment

Participants completed unweighed food diaries, including all food and beverages consumed both inside and outside the home for three or four consecutive days. This process is described in detail elsewhere [42]. Participants also recorded where the food was eaten, for example at home, in a restaurant or café, or at work. This variable included a specific category for food eaten at work but brought from home.

### Characterisation of food-related variables

Food-related variables – proportion of home-prepared food and dietary quality, as well as other aspects of diet such as daily intake of food groups, energy and macro- and micronutrients – were derived from food diaries. The first two variables were derived in order to classify participants as being either in the high or low home preparation group. Further food-related variables were derived in order to characterise dietary intake in greater detail.

#### Proportion of energy from home-prepared food

Food items listed in food diaries were classified by the authors as either requiring or not requiring home preparation. All foods were classified as home-prepared except those listed in Table 1. Foods which should not be classified as being home-prepared were decided by the authors a priori.

**Table 1:** Foods not classified as home-prepared

▪ Foods prepared and eaten outside the home (e.g. food eaten in a restaurant or café) ▪ Foods prepared outside the home and eaten in the home (e.g. takeaway and delivery foods) ▪ Foods eaten as purchased (e.g. crisps, sweets, granola bars, juice and soft drinks, store-bought sandwiches, prepared and whole pieces of fruit) ▪ Foods requiring the application of heat or the addition of hot water but no other preparation (e.g. frozen and refrigerated ready meals, tinned soup, instant noodles, instant oats) ▪ Foods involving the combination of several components by the participant, but each component required minimal preparation (e.g. a bowl of cereal, a ham or cheese sandwich)	

Definitions of ‘cooking’ have been discussed extensively and remain contested [21, 43, 44], with many definitions not deeming the application of heat to be a necessary part of this process [44, 45]. As a result ‘home food preparation’ and ‘home-prepared food’ seem more accurate and are the concepts deployed here. Different, but related, conceptualisations exist, such as food ‘prepared from scratch’ [46], or food that is not ‘from outside the home’ [7]. The conceptualisation of home-prepared food used here reflects several conceptions of ‘cooking’, or home food preparation, drawn from qualitative studies [47, 48] as well as behaviours which are habitually enquired about in studies of ‘cooking’, such as blending, mixing, boiling, chopping, roasting and pan frying [49]. From this conceptualisation of home food preparation, a set of behaviours, we defined foods which we would deem to be home-prepared as being the products of these behaviours.

**Fig. 1:**
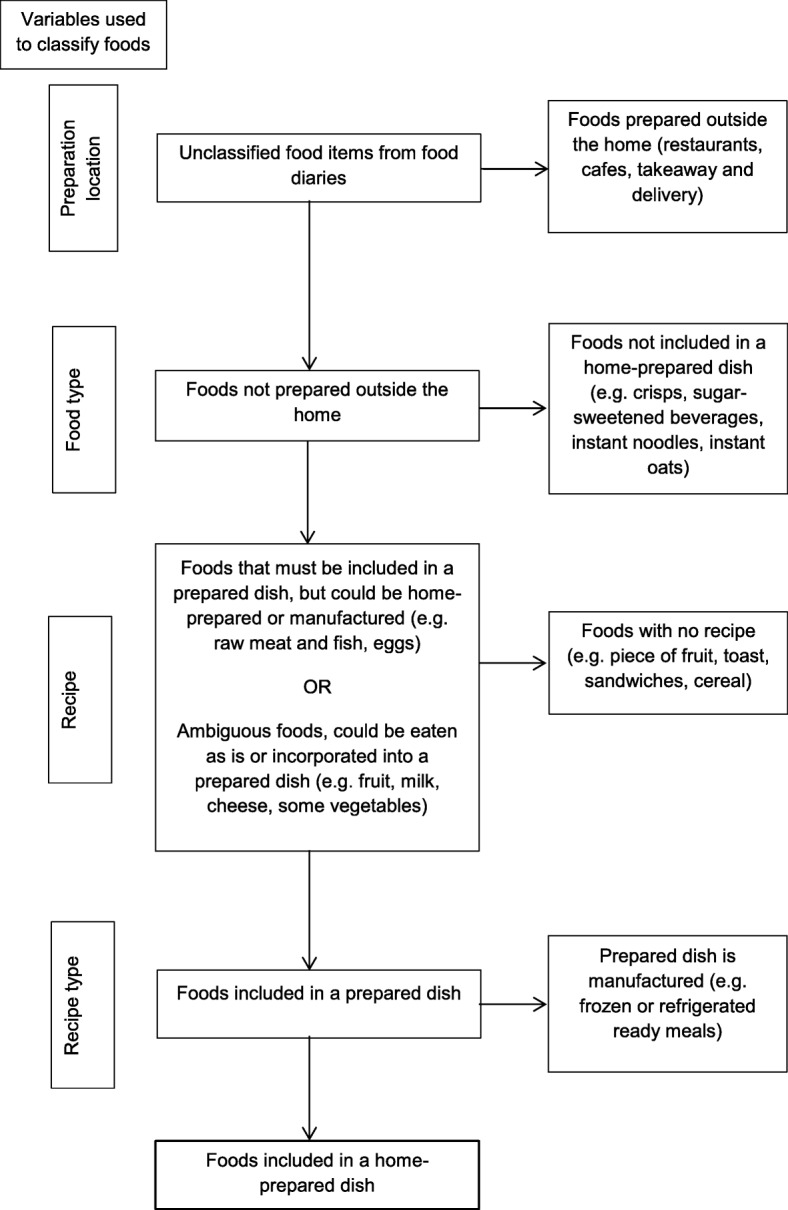
Flow diagram for classification of foods as being home-prepared

Food classification was carried out using food diary variables as illustrated in Fig. 1, with foods which were not classified as home-prepared being successively removed until only food included in home-prepared dishes remained. The proportion of energy from home-prepared food was then calculated for each participant by summing the energetic content of foods classified as home-prepared and dividing them by the participant’s total energy intake. Participants were then separated into tertiles based on this proportion. Individuals in the highest tertile for proportion of energy from home-prepared foods were categorised as belonging to the high home preparation group, while those in the lowest tertile were categorised as belonging to the low home preparation group.

#### Dietary quality

Dietary quality was determined by quantifying accordance to the Dietary Approaches to Stopping Hypertension (DASH) dietary pattern using a method adapted for use with NDNS [50] from an existing index [51]. The DASH diet has been shown to lower blood pressure [52] and reduce low-density lipoprotein cholesterol levels, [52] as well as being associated with a lower risk of stroke and coronary heart disease [51]. This score is based on food and nutrients emphasised or minimised in the DASH diet, and has eight components: high intake of fruits, vegetables, nuts and legumes, low-fat dairy products, and whole grains; and low intake of sodium, red and processed meats, and non-extrinsic milk sugars; all adjusted for total energy intake. The score is adjusted for overall energy intake. Components are evenly weighted, and three components (sodium, sugar, and red and processed meats) are reverse-scored, so that higher consumption would lower an individual’s DASH score.

Participants were separated into tertiles by DASH score. Participants in the highest tertile were categorised as high-DASH.

#### Intake of energy, macronutrients and micronutrients

Mean daily intake of energy was estimated by the NDNS team using food diaries, as were daily intakes of several macro- and micronutrients: fat, saturated fat, protein, carbohydrate, sugar, and sodium, a process described in detail elsewhere [42]. These nutrients make up mandatory nutrition labelling in the UK [53]. Intake was categorised as meeting or not meeting relevant UK dietary guidelines, [54, 55] except in the case of carbohydrates. Current UK recommendations suggest a population mean of approximately 50% of total energy from carbohydrate, but note that total carbohydrate intake does not appear to be associated with health outcomes, as it is composed of different nutrients such as fibre, sugar and starches, which may have a variety of impacts [56]. Therefore, carbohydrate intake was described in all groups, but adherence to a particular recommendation was not defined.

Daily intakes of other nutrients were also estimated by the NDNS team using food diaries [42]. Where UK guidelines existed, [54] adherence to these guidelines was also determined. Nutrients included were: fibre, thiamine, riboflavin, niacin, vitamin B6, vitamin B12, folate, vitamin C, vitamin A, calcium, phosphorus, magnesium, zinc, selenium, iodine, iron, chloride, vitamin E, copper, manganese, biotin, and pantothenic acid. Nutrients derived from supplements were not included in the data presented here.

#### Intake of food groups

Daily intakes of the main food groups determined by NDNS were calculated using food diaries. Where possible similar groups of food were collapsed (e.g. 1% fat milk, skimmed milk and semi-skimmed milk).

### Prevalence of overweight and obesity

Interviewers collected measurements of height and weight from NDNS participants using standard protocol. These measures were used to calculate BMI, which was categorised as overweight/obese (BMI ≥25 kg/m^2^), or not.

### Socio-demographic variables

Socio-demographic variables considered include age, sex and ethnicity (categorised as white or not due to the high proportion of white participants in NDNS) were determined using self-reported survey responses, as were the presence of a child under 16 years of age in participant households. Socioeconomic position was also assessed using self-reported survey responses, and was characterised using a range of markers: occupation (professional/other), education (degree level or above/other), and annual income equivalised for household composition (above or below £35,000).

### Analysis

The demographic and socioeconomic characteristics of individuals in the high-home preparation and low home-preparation groups were described. The statistical significance of differences between groups was tested using either a linear or logistic regression as appropriate, mutually adjusted for all other socio-demographic variables.

Overall dietary characteristics were examined in two ways. First, the high home preparation and low home preparation groups were compared in terms of DASH score, proportion of energy from home-prepared food, energy intake, and adherence to dietary guidelines for macro- and micronutrients. Prevalence of overweight or obesity was also compared across groups. Second, the groups were compared in terms of their intake of each of the food groups or nutrients that make up each of the eight components of the DASH diet and index: low-fat dairy, whole grain, fruit, vegetables, nuts and legumes, sodium, sugars, and red and processed meats. In both cases, the statistical significance of differences between groups was tested using either a linear or logistic regression as appropriate, adjusted for all socio-demographic variables.

In addition, food-level differences between home preparation groups were then assessed through an examination of the food group codes provided by NDNS. Due to the high proportion of individuals who did not consume many of the food groups over the course of the recorded days, this was done in two steps. First, the proportion of individuals consuming any amount from each food groups was calculated for both the high home preparation and the low home preparation groups. Differences in these proportions were tested using logistic regression. Second, the median quantity of each food group consumed by consumers of those food groups was determined. Differences between home preparation groups in these quantities were tested using linear regression. All regressions in food-level analyses were adjusted for all socio-demographic variables.

All analyses were conducted using Stata (version 14; Stata Corp.). Alpha-level of 0.05 was used throughout.

## Results

Overall, 54% (*N* = 12,070) of individuals selected to take part in NDNS provided useable food diaries (three or four complete days), including 6364 adults [39, 40, 57]. Adult participants classified by tertile of DASH score and proportion of energy derived from home-prepared food are displayed in Table 2.

**Table 2:** Adult NDNS participants by tertile of DASH score and proportion of energy derived from home-prepared food n (% of adult study sample)

DASH score	Proportion of energy from home-prepared food			Total
	Low	Medium	High	
Low	1095 (17.2)	836 (13.1)	679 (10.7)	2610 (41.0)
Medium	703 (11.1)	697 (11.0)	713 (11.2)	2113 (33.2)
High	442 (7.0)	578 (9.1)	621 (9.8)	1641 (25.8)
Total	2240 (35.2)	2111 (33.2)	2013 (31.6)	6364 (100.0)

The analytic sample used in this study therefore included 1063 participants (16.7% of adult NDNS sample): 621 (9.8%) participants in the high home preparation group, and 442 (7.0%) participants in the low home preparation group. While NDNS is a nationally representative sample, the analytic sample differs from the rest of the NDNS sample, notably in having a higher socioeconomic position, as well as being older, less likely to be male or white, and less likely to have a child aged under 16 living at home (Table 3 in Appendix).

Table 3 presents sample demographic and socioeconomic characteristics for individuals in the high and low home preparation groups. Table 4 shows that, after adjustment for all other sociodemographic variables, individuals in the low home preparation group were more likely to be older and white, and less likely to have a degree level education relative to the high home preparation group.

**Table 3:** Demographic and socioeconomic characteristics for high and low home preparation groups

Characteristic	High DASH			
	High home preparation	Low home preparation	Total	OR/regression coefficient (95 %CI)^a, b^
n	621	442	1063	
Demographic				
Age (mean (95% CI))	51.0 (49.3, 52.6)	54.6 (52.7, 56.5)	52.4 (51.2, 53.7)	*3.02 (0.47, 5.57)* ^c^
Sex (% male)	39.8	45.7	42.2	0.81 (0.58, 1.10)
Ethnicity (% white)	76.7	90.4	82.8	*0.42 (0.25, 0.73)*
Children (% with a child aged < 16)	31.2	23.9	28.3	0.96 (0.62, 1.48)
Socioeconomic				
Education (% degree)	41.9	34.0	38.7	*0.69 (0.48, 0.99)*
Equivalised income (% > £35,000)	33.5	39.7	36.0	1.31 (0.90, 1.93)
Occupation (% professional)	50.9	55.7	52.8	1.17 (0.82, 1.68)

^a^Mutually adjusted for socio-demographic variables excluding the dependent variable: age, sex, ethnicity, children, education, income and occupation

^b^Odds ratios (95%CI), except for in the case of age

^c^This number represents a regression coefficient (95% CI), as age was analysed as a continuous variable

Note: Italics indicate statistical significance (*p* < 0.05)

Table 4 presents an overview of dietary characteristics of those in the high and low home preparation groups. Table 4 shows that, after adjustment for socio-demographic characteristics, both groups achieved the same levels of DASH adherence, and showed no significant differences in their mean daily energy intake (kcal) or their prevalence of overweight and obesity, despite proportion of energy they derive from home-prepared food being substantially and significantly different. At a nutrient level, however, some differences emerged. In the low home preparation group, a smaller proportion of participants adhered to dietary guidelines for sugar and sodium; but there were no between-group differences in proportion adhering to guidelines on fat, saturated fat and protein.

**Table 4:** Dietary characteristics and prevalence of overweight or obesity for high and low home preparation groups

Characteristic	High DASH			
	High home preparation	Low home preparation	Total	Regression coefficient (95 %CI)^a^
DASH score (Median (IQR))				
	30 (29, 32)	30 (29, 32)	30 (29, 32)	−0.32 (− 0.71, 0.07)
% of total energy from home-prepared food (Mean (95% CI))				
	41.8 (40.8, 42.7)	15.4 (14.8, 15.9)	31.2 (29.9, 32.5)	*−0.26 (− 0.27, − 0.25)*
Mean daily energy intake kcal (Mean (95% CI))				
	1772 (1720, 1825)	1861 (1804, 1918)	1808 (1769, 1847)	45.6 (− 25.8, 117.1)
Prevalence of obesity/overweight (% obese or overweight (≥25 kg/m^2^))				
	52.6	55.8	53.9	1.07 (0.78, 1.46)
Mean daily nutrient intake: % meeting guidelines	High home preparation	Low home preparation	Total	OR (95 %CI)^a^
Fat (< 35% energy)	58.8	60.0	59.3	1.07 (0.77, 1.49)
Saturated fat (< 11% energy)	45.1	38.1	42.3	0.85 (0.61, 1.18)
Protein (45–56 g)^b^	92.4	90.8	91.8	0.53 (0.28, 1.01)
Sugar (< 11% energy)	79.1	59.0	71.1	*0.39 (0.27, 0.55)*
Sodium (< 1600 mg)	36.0	26.3	32.1	*0.71 (0.51, 1.00)*
Carbohydrate	49.3 (48.4, 50.2)	48.5 (47.9, 49.1)	48.0 (47.3, 48.7)	N/A

^a^Mutually adjusted for age, sex, ethnicity, children, education, income and occupation

^b^Dependent on body mass

Note: Italics indicate statistical significance (*p* < 0.05)

Information about adherence to micronutrient guidelines can be found in Table 6 in Appendix. The low home preparation group had a higher prevalence of individuals meeting guidelines for riboflavin, folate and calcium, while the high home preparation group saw more participants meeting guidelines for vitamin A, zinc and selenium. There were no significant differences in adherence to fibre guidelines.

Table 5 presents the daily quantity consumed of each of the eight food groups and nutrients that make up the DASH index.

**Table 5:** Daily quantity of each DASH component consumed for high and low home preparation groups (Median (IQR))

DASH Component (median (IQR))	High DASH			
	High home preparation	Low home preparation	Total	Regression coefficient (95 %CI)^a^
Low-fat dairy (g)	186.9 (102.8, 283.0)	237.5 (143.8, 325.6)	207.5 (120.0, 305.0)	*24.6 (1.7, 47.5)*
Whole grain (g)	73.8 (46.0, 125.0)	86.0 (53.9, 128.0)	78.8 (48.8, 125.8)	2.2 (−6.8, 11.2)
Fruit (g)	193.8 (129.0, 297.1)	218.6 (138.8, 342.5)	201.8 (131.9, 312.6)	*30.81 (5.51, 56.1)*
Vegetables (g)	220.0 (167.2, 295.5)	175.7 (123.8, 225.4)	195.6 (143.7, 264.6)	*−52.6 (−66.7, 38.6)*
Nuts & legumes (g)	24.1 (0.8, 52.5)	26.4 (3.0, 52.5)	24.6 (0.9, 52.5)	−0.4 (−7.7, 7.0)
Sodium (mg)	1855.4 (1469.8, 2361.0)	1963.5 (1571.4, 2436.2)	1908.5 (1518.1, 2388.2)	*107.8 (13.8, 201.8)*
Sugars (g)	34.0 (20.7, 51.8)	44.3 (27.9, 65.4)	39.5 (23.1, 57.3)	*11.6 (7.5, 15.6)*
Red & processed meats (g)	39.0 (10.0, 73.8)	28.3 (1.5, 53.6)	33.0 (5.5, 65.0)	*−10.4 (− 16.6, −4.3)*

^a^Mutually adjusted for age, sex, ethnicity, children, education, income and occupation

Note: Italics indicate statistical significance (*p* < 0.05)

Differences in quantities suggest that the high and low home preparation groups are achieving this measure of high dietary quality through different foods and nutrients. The low home preparation group consumed more fruit and low fat dairy products, but also more sugar and sodium. The high home preparation group consumed more vegetables than their low home preparation counterparts, but also more red and processed meat.

More granular, food-level analysis of participant diets can be found in Table 8 in Appendix. These results mirror those displayed in Table 5: the low home preparation group consumed more low-fat dairy foods such as yoghurt and milk, while the high home preparation group consumed more vegetables. The low home preparation group consumed more whole grain foods requiring limited preparation, such as wholemeal bread and high fibre breakfast cereals. They also consumed a larger number of sweet things, such as sugar-sweetened beverages, biscuits and chocolates, as well as more crisps and salty snacks, mirrored by the higher levels of sugar and sodium in their diets. The high home preparation group ate more beef and lamb, contributing to a higher overall consumption of red and processed meat. Some results from the food-level analysis were not captured in Table 5, because the DASH index does not take them into account. For example, the high home preparation group also ate more eggs, chicken and fish, while the low home preparation group drank more wine and beer.

## Discussion

### Principal findings

This is the first analysis we are aware of to explore whether substantial consumption of home-prepared food is necessary in order to achieve a high quality diet. We found that it is not: 7% of adult NDNS participants were in the top tertile for dietary quality as indicated by DASH score, while being in the bottom tertile of proportion of energy derived from home-prepared foods. While all study participants were in the highest tertile of DASH score, there was also no significant difference between the median DASH scores of the high and low home preparation groups, in their energy intakes, nor in the prevalence of overweight or obesity between groups.

Relative to their counterparts with a similar dietary quality who relied more heavily on home-prepared food, individuals in the low home preparation group are likely to be older, more likely to be white and less likely to have a degree level education. There are no significant differences in income or occupational grade between the two groups, although both groups were significantly more affluent in terms of education, income and occupation than the NDNS sample as a whole.

### Strengths and weaknesses

From a socio-demographic perspective, the analytic sample used in this study was significantly different to the nationally representative NDNS sample (see Table 3 in Appendix). This was due to the research question, which demanded that only individuals in the top tertile of dietary quality be included. As previous studies have shown, individuals with high dietary quality tend to have certain socio-demographic traits, such as being older and more affluent, [58, 59] a pattern which is reflected here.

The positive impact of the DASH diet on hypertension as well as on other chronic diseases has been repeatedly demonstrated, [51, 52] and the DASH index used in this study is in line with that used in epidemiological studies that have reported these associations [51]. As a marker of dietary quality, it is very well-evidenced. However, these studies relate the positive health associations of DASH to ‘DASH adherence’, defined as the top quintile of a population’s DASH score. In this study, a wider definition was used in order to increase sample size, and participants in the top tertile of DASH were defined as having a relatively high dietary quality.

In addition, DASH does not take into account all of the foods that individuals may eat. The fact that some food-level differences are not captured by an examination of DASH score components highlights this. For example, the high home preparation group ate more fish and eggs, while the low home preparation group consumed more wine and beer.

Finally, diet-related disease may be caused by an excess consumption of energy, regardless of dietary quality. However, the DASH score does make food group consumption relative to the overall energetic content of the diet. In addition, the two groups under study did not differ in terms of energy intake.

Food-related measures were derived from food diaries, which were unweighed and self-reported. Some evidence suggests that food diaries are a more accurate measure of dietary intake than other common measures such as food frequency questionnaires [60]. However, misreporting in self-measured dietary instruments is a well-documented limitation, [61] and biomarker analysis of a sub-group of NDNS suggests participants underreport the energy they consume, [57] which may explain the surprisingly low average calorie intake in participants included in this study (Table 5). While this introduces error, it is not clear whether the two groups under study might misreport in systematically different ways.

This study took a novel approach to quantifying the proportion of home-cooked food in participants’ diets. Previous studies and surveys have approached this by asking participants how often they cook, or how often they eat home-prepared or home-cooked foods [62]. These methods are subject to some limitations. The social desirability of cooking and home-cooked food [63] may introduce bias into participant responses to these sorts of questions. In addition, qualitative studies [45] suggest that there is some disagreement among study participants as to what constitutes home-cooked or –prepared food, meaning that the same response may mean different things to different people. While the classification of foods as home-prepared or not used in the current study may be somewhat arbitrary, and is certainly debatable, it has the advantage of being independent of participant interpretations of questions concerning cooking frequency or frequency of preparing meals from scratch.

### Implications of the findings

Previous research has concluded that more frequent consumption of home-prepared food is associated with a higher dietary quality [62]. While this may also be the case in the NDNS sample, the public health community currently lacks an effective method for changing home food preparation practices, as discussed in the introduction. While not discounting the existing work on this subject, it is difficult to see how to move forward with these findings. This study has instead explored individuals who do the unexpected, by eating healthily with minimal energetic contribution from home-prepared foods.

The finding that substantial consumption of home-prepared food is not necessary to achieve high dietary quality suggests that cooking skills interventions and dietary guidelines which emphasise home food preparation as being necessary to a healthy diet may be inappropriate. While home food preparation may be a useful practice for some to achieve greater dietary quality, it does not appear to be a necessary one. Recognising that people can have high quality diets with or without cooking and supporting them to eat healthily regardless seems important.

Examining the dietary composition of the low home preparation group might shed some light on how to support a healthy diet in people who do not eat much home-prepared food. The results of this study suggest that it is their high intake of sugar and sodium which must be addressed. The food-level analysis suggests that this intake may be driven by a higher consumption of prepared foods, such as biscuits, chocolate and candy, soft drinks, and crisps and other snacks. Sugar and salt reduction programmes are already under way in the UK, [64, 65] as well as globally [66, 67]. This higher intake of sugar and sodium could be addressed through further reformulation of these prepared foods. In addition, ways of increasing the availability of vegetables requiring little home preparation might be explored, such as increasing the servings of vegetables in prepared meals.

Although their diets are less healthy on some dimensions, most notably in the higher sugar and sodium content of their diet, participants in the low home preparation group are still achieving the same overall dietary quality as indicated by DASH score. This reflects that the DASH score is made up of several evenly weighted components, meaning that a given DASH score could reflect different combinations of healthy and less healthy foods.

Similar scores overall may also reflect the fact that DASH is a relative measure, and that the DASH scores of the participants discussed here were derived from an analysis of the complete adult NDNS sample, which included participants with a lower DASH score whose diets were not analysed in this study (Table 9 in Appendix). Although the low home preparation group were eating more of some ‘unhealthy’ foods than the high home preparation groups, their quantities were relatively similar in comparison to the quantities of the wider (lower-DASH) sample. This highlights the fact that a diet that is ‘healthy’ relative to population levels can still include some ‘unhealthy’ things.

The affluent nature of the analytic sample, which reflects a socioeconomic gradient in diet quality reported by many studies [58, 59] may limit some of the implications that can be drawn from this study. For example, it was noted that there are no significant differences in income between the low home preparation and the high home preparation groups. This might suggest that there is no additional cost involved in eating healthily without much home-prepared food. However, when we note that the analytic sample had a significantly higher prevalence of high-income individuals than the NDNS sample as a whole, it seems plausible that for individuals in the analytic sample cost is not a significant barrier, and dietary practices are driven more by other factors such as time or taste. While it may be possible to eat healthily without eating much home-prepared food, doing so may be more expensive.

### Unanswered questions and future research

We require a better understanding of the conditions necessary to achieve a high quality diet while eating low amounts of home-prepared food. The relatively high socioeconomic position of the analytic sample may mean that this group has more financial resources, or access to a specific array of food outlets due to neighbourhood food environment. Other conditions may also be necessary, such as food and nutrition knowledge, motivation, kitchen facilities, or access to a car. Further research may allow us to understand how practicable the high DASH low home preparation pattern is for the wider population, and what interventions might be carried out to make it practicable for larger numbers.

While cooking skills and practices have been discussed extensively, [21, 45, 68] eating healthily without relying on home-prepared food may also rely on its own non-cooking set of skills and practices. These could also be an interesting matter for research, although they may be as resistant to change through education interventions as cooking practices appear to be.

The use of food diaries to characterise dietary intake using related concepts such as food ‘prepared from scratch’ could be further investigated. The way these concepts relate to indices of dietary quality, nutritional intake, socio-demographic characteristics and health outcomes could be analysed.

Finally, a mirror analysis might be carried out which investigates home-prepared food consumption in a sample with low dietary quality.

## Conclusion

This study suggests that consuming a substantial amount of home-prepared food is not necessary to achieve high dietary quality: a set of food practices are present in a sizable proportion of the population which allow individuals to achieve a high dietary quality while relying minimally on home-prepared foods. However, participants included in this study were significantly more affluent than the nationally representative sample from which they were drawn, suggesting thatthe practices in which these individuals engage may be dependent on socioeconomic position. This bears further inquiry.

The low home preparation group consumed more fruit and low-fat dairy products, and less red meat than the high home preparation group, but also more sugar and sodium, highlighting a need for further reduction of sugar and sodium in prepared foods.

The public health community should recognise the existence of a set of food practices which allows individuals to achieve a healthy diet with little contribution from home-prepared food, and make space for it in the design of their policies and interventions.

## Appendix

**Table 6:** Demographic and socioeconomic characteristics of analytic sample and rest of NDNS sample

Characteristic	High DASH analytic sample	Rest of sample	Total	Coefficient (95 %CI)/ x^2^	*p* value
n	1063	5301	6364		
Demographic					
Age (mean (SD))	52.4 (51.2, 53.7)	47.0 (46.3, 47.8)	48.0 (47.3, 48.6)	5.4 (4.0, 6.8)	< 0.01
Sex (% male)	42.2	50.0	48.6	22.8	< 0.01
Ethnicity (% white)	82.8	90.4	88.9	74.9	< 0.01
Children (% with a child aged < 16)	28.3	32.3	31.6	18.6	< 0.01
Socioeconomi*c*					
Education (% degree)	38.7	22.6	25.5	127.4	< 0.01
Equivalised income (% > £35,000)	36.0	26.8	28.5	38.4	< 0.01
Occupation (% professional)	52.8	40.2	42.5	60.8	< 0.01

Note: Italics indicate statistical significance (*p* < 0.05)

**Table 7:** Adherence to nutrient guidelines for high and low home preparation groups

Characteristic	High DASH				*p* value
	High home preparation	Low home preparation	Total	OR (95% CI)^a^	
Daily nutrient intake					
% meeting guidelines					
Thiamin	96.5	98.0	97.1	1.4 (0.4, 4.6)	0.63
Riboflavin	74.5	86.7	79.4	1.7 (1.1, 2.7)	*0.02*
Niacin	99.9	100.0	99.9	N/A	
Vitamin B6	88.5	90.9	89.4	1.2 (0.7, 2.2)	0.54
Vitamin B12	96.2	96.1	96.1	0.6 (0.2, 1.6)	0.31
Folate	78.6	89.0	82.8	2.0 (1.2, 3.3)	*< 0.01*
Vitamin C	94.8	95.3	95.0	1.0 (0.5, 2.0)	0.98
Vitamin A	76.8	68.9	73.7	0.6 (0.4, 0.9)	*0.02*
Calcium	63.2	77.6	69.0	1.6 (1.1, 2.3)	*0.01*
Phosphorus	99.7	99.8	99.7	1.2 (0.1, 12.6)	0.88
Magnesium	54.6	57.3	55.7	1.0 (0.8, 1.4)	0.87
Potassium	28.7	36.3	31.7	1.2 (0.8, 1.7)	0.42
Iron	61.9	69.5	64.9	1.0 (0.7, 1.5)	0.87
Zinc	64.6	58.3	62.1	0.7 (0.5, 1.0)	*0.03*
Selenium	25.3	15.3	21.3	0.6 (0.4, 0.8)	*< 0.01*
Iodine	60.2	68.8	63.7	1.1 (0.8, 1.6)	0.47
Chloride	100.0	100.0	100.0	N/A	
Vitamin E	28.1	27.8	27.9	1.0 (0.7, 1.5)	0.80
Copper	55.2	52.0	53.9	0.8 (0.6, 1.1)	0.21
Manganese	97.0	98.3	97.5	1.7 (0.5, 5.1)	0.38
Biotin	22.0	19.8	21.1	0.7 (0.5, 1.1)	0.15
Pantothenic acid	48.4	52.1	49.9	1.0 (0.7, 1.3)	0.78
Fibre (≥30 g)	2.6	1.3	2.1	0.5 (0.1, 1.7)	0.26

^a^Adjusted for age, sex, ethnicity, children, education, income and occupation

Note: Italics indicate statistical significance (*p* < 0.05)

**Table 8:** Percentage of individuals who eat foods from each food group by level of home-prepared food

Food group	High DASH			OR (95% CI)^a^	*p*-value
	High home preparation	Low home preparation	All		
Reduced fat milk	83.9	94.5	88.1	2.7 (1.5, 4.7)	*< 0.01*
Whole milk	20.3	8.9	15.7	0.5 (0.3, 0.7)	*< 0.01*
Other milk and cream	42.9	32.4	38.7	0.6 (0.4, 0.8)	*< 0.01*
Yogurt, fromage frais and dairy desserts	56.1	65.9	60.0	1.5 (1.1, 2.0)	*0.02*
Butter	23.1	30.0	25.8	1.6 (1.1, 2.2)	*0.02*
Reduced fat spread	53.2	65.6	58.2	1.6 (1.1, 2.2)	*< 0.01*
PUFA margarines and oils	12.1	8.0	10.5	0.7 (0.4, 1.3)	0.28
Other margarine, fats and oils	31.0	23.9	28.1	0.7 (0.5, 1.0)	0.08
Cheese	66.1	75.4	69.8	1.4 (1.0, 2.0)	0.06
Eggs and egg dishes	71.2	55.6	65.0	0.5 (0.3, 0.7)	*< 0.01*
Brown bread	79.9	85.0	82.0	1.4 (1.0, 2.2)	0.08
White bread	59.8	65.8	62.2	1.3 (1.0, 1.8)	0.09
Other bread	6.4	11.0	8.2	2.1 (1.1, 3.7)	*0.02*
High fibre breakfast cereals	56.3	71.1	62.2	1.7 (1.2, 2.3)	*< 0.01*
Other breakfast cereals	23.1	30.0	25.8	1.6 (1.1, 2.2)	*0.02*
Pasta, rice and other cereals	45.9	48.5	46.9	0.8 (0.6, 1.1)	0.26
Nuts and seeds	48.7	45.0	47.2	0.9 (0.6, 1.2)	0.44
Salad and other raw vegetables	93.4	91.9	92.8	0.8 (0.5, 1.5)	0.55
Vegetables not raw	100.0	98.0	99.2	N/A	
Fruit	98.2	98.2	98.2	0.8 (0.3, 2.4)	0.70
Fruit juice	51.9	53.1	52.4	1.2 (0.8, 1.6)	0.36
Smoothies 100% fruit and/or juice	1.7	2.0	1.9	1.4 (0.4, 4.7)	0.56
Biscuits, chocolate and candy	74.8	84.0	78.3	1.7 (1.2, 2.6)	*< 0.01*
Ice cream	16.9	27.0	21.0	1.8 (1.2, 2.6)	*< 0.01*
Puddings	22.1	20.8	21.6	0.8 (0.6, 1.2)	0.37
Buns, cakes, pastries and fruit pies	47.2	64.1	53.9	1.9 (1.3, 2.6)	*< 0.01*
Sugars, preserves and sweet spreads	75.8	70.5	73.7	0.8 (0.6, 1.2)	0.25
Crisps and savoury snacks	29.4	39.4	33.4	1.6 (1.2, 2.2)	*< 0.01*
Oily fish	42.9	36.8	40.5	0.6 (0.5, 0.9)	*0.01*
White fish breaded or fried	14.8	20.6	17.1	1.4 (1.0, 2.1)	0.07
Other fish dishes (incl. White fish and shellfish)	46.2	46.8	46.4	0.9 (0.7, 1.3)	0.65
Bacon and ham	3.8	6.0	4.7	2.2 (1.1, 4.4)	*0.03*
Pork and dishes	13.9	24.4	18.1	1.8 (1.2, 2.6)	*< 0.01*
Sausages	18.7	19.4	19.0	0.9 (0.6, 1.4)	0.78
Beef, veal and dishes	43.8	33.2	39.5	0.6 (0.4, 0.8)	*< 0.01*
Lamb and dishes	16.4	11.4	14.4	0.7 (0.5, 1.1)	0.17
Liver and dishes	6.4	7.7	6.9	1.1 (0.6, 2.1)	0.77
Chicken and turkey dishes	66.1	62.2	64.5	0.8 (0.6, 1.2)	0.31
Coated chicken	3.8	6.0	4.7	2.2 (1.1, 4.4)	*0.03*
Burgers and kebabs	4.7	4.9	4.8	1.0 (0.4, 2.1)	0.90
Meat pies and pastries	5.9	14.3	9.3	2.4 (1.4, 4.1)	*< 0.01*
Other meat and meat products	45.9	48.5	46.9	0.8 (0.6, 1.1)	0.26
Chips, roasted and fried potatoes	42.7	49.9	45.6	1.3 (0.9, 1.7)	0.14
Other potato dishes	76.4	84.6	79.7	1.4 (0.9, 2.2)	0.13
Artificial sweeteners	11.1	15.6	12.9	1.4 (0.9, 2.1)	0.14
Dietary supplements	36.8	40.7	38.4	1.1 (0.8, 1.5)	0.67
Tea, coffee and water	100.0	99.5	99.8	N/A	
Soft drinks low calorie	24.5	33.4	28.0	1.6 (1.1, 2.4)	*0.01*
Soft drinks not low calorie	24.0	38.1	29.6	2.0 (1.4, 2.9)	*< 0.01*
Beer, lager, cider and perry	22.0	27.5	24.2	1.2 (0.8, 1.8)	0.27
Wine	43.0	43.6	43.2	0.8 (0.6, 1.2)	0.32
Spirits and liqueurs	9.3	16.0	12.0	1.5 (0.9, 2.5)	0.13
Miscellaneous	98.2	94.2	96.6	0.3 (0.1, 0.7)	*< 0.01*

^a^Adjusted for age, sex, ethnicity, children, education, income and occupation

Note: Italics indicate statistical significance (*p* < 0.05)

**Table 9:** Median (IQR) grams of food eaten daily by high-DASH consumers of those foods

Food group	High DASH			Coefficient (95% CI)^a^	*p*-value
	High home preparation	Low home preparation	All		
Reduced fat milk	172.5 (88.1, 253.5)	190.0 (105.0, 292.5)	178.8 (96.3, 267.5)	12.4 (−10.0, 34.9)	0.28
Whole milk	52.5 (9.5, 159.2)	25.0 (10.0, 149.3)	50.0 (9.5, 159.2)	−5.9 (− 13.6, 1.8)	0.13
Other milk and cream	21.0 (9.6, 62.5)	12.4 (7.5, 30.0)	16.3 (7.5, 48.8)	−16.7 (−36.2, 2.7)	0.09
Yogurt, fromage frais and dairy desserts	60.0 (31.3, 93.8)	68.1 (43.8, 100.0)	62.5 (32.5, 100.0)	13.1 (3.0, 23.3)	*0.01*
Butter	15.0 (7.5, 27.5)	20.0 (10.0, 30.0)	15.0 (9.0, 30.0)	2.6 (−1.4, 6.7)	0.20
Reduced fat spread	8.5 (4.3, 14.6)	11.2 (6.8, 17.0)	10.0 (5.0, 16.3)	2.1 (0.9, 3.4)	*< 0.01*
PUFA margarines and oils	1.9 (0.7, 7.8)	1.4 (0.4, 2.5)	1.5 (0.7, 4.5)	−1.3 (−3.1, 0.5)	0.16
Other margarine, fats and oils	2.9 (1.5, 6.2)	1.4 (0.7, 3.0)	2.6 (1.1, 4.8)	−1.4 (−2.3, −0.5)	*< 0.01*
Cheese	18.3 (10.0, 30.6)	18.8 (10.0, 32.3)	18.5 (10.0, 31.3)	0.2 (−2.7, 3.0)	0.91
Eggs and egg dishes	30.0 (15.0, 45.0)	28.5 (14.3, 44.3)	28.5 (15.0, 45.0)	−4.8 (−8.7, −1.0)	*0.01*
Brown bread	49.7 (27.6, 72.6)	59.2 (33.5, 89.5)	52.2 (30.0, 82.5)	10.4 (5.3, 15.4)	*< 0.01*
White bread	30.3 (18.0, 52.8)	36.0 (20.0, 73.0)	32.4 (18.7, 61.9)	10.5 (4.8, 16.2)	*< 0.01*
Other bread	24.5 (14.8, 38.6)	29.3 (20.6, 43.0)	25.9 (20.0, 38.8)	7.9 (−4.9, 20.7)	0.23
High fibre breakfast cereals	29.3 (15.0, 54.0)	36.6 (20.0, 66.0)	32.5 (17.5, 59.4)	3.2 (−4.4, 10.7)	0.41
Other breakfast cereals	15.0 (7.5, 27.5)	20.0 (10.0, 30.0)	15.0 (9.0, 30.0)	2.6 (−1.4, 6.7)	0.20
Pasta, rice and other cereals	12.5 (6.3, 25.8)	12.5 (5.8, 24.8)	12.5 (6.3, 25.0)	−1.3 (−3.7, 1.2)	0.32
Nuts and seeds	12.5 (5.6, 25.0)	9.7 (6.3, 20.9)	12.5 (5.9, 24.5)	−0.9 (−5.9, 4.2)	0.74
Salad and other raw vegetables	56.4 (24.3, 94.6)	63.6 (27.0, 103.4)	60.1 (26.3, 98.6)	6.7 (−1.3, 14.6)	0.10
Vegetables not raw	190.2 (131.9, 259.4)	139.2 (95.0, 183.3)	163.1 (116.3, 232.3)	−52.3 (−63.7, −40.9)	*< 0.01*
Fruit	196.0 (132.2, 301.3)	219.4 (145.2, 348.8)	203.9 (134.9, 314.6)	7.5 (−8.0, 23.0)	0.34
Fruit juice	77.5 (31.3, 150.0)	112.5 (50.0, 175.0)	93.8 (37.5, 150.0)	32.3 (14.5, 50.2)	*< 0.01*
Smoothies 100% fruit and/or juice	66.3 (66.3, 198.8)	66.3 (43.5, 95.4)	66.3 (66.3, 95.4)	23.0 (−119.7, 165.7)	0.73
Biscuits, chocolate and candy	14.8 (8.1, 25.0)	20.4 (10.0, 40.0)	17.5 (8.8, 32.8)	11.3 (8.0, 14.6)	*< 0.01*
Ice cream	30.0 (21.3, 50.0)	27.5 (16.3, 50.0)	30.0 (20.0, 50.0)	0.5 (−4.6, 5.5)	0.85
Puddings	35.5 (22.5, 53.1)	27.5 (16.9, 50.0)	30.0 (20.0, 50.0)	−0.8 (− 11.2, 9.7)	0.89
Buns, cakes, pastries and fruit pies	24.0 (14.0, 41.3)	30.0 (15.8, 54.5)	27.5 (15.0, 48.0)	5.8 (1.9, 9.7)	*< 0.01*
Sugars, preserves and sweet spreads	12.0 (5.4, 20.2)	9.0 (3.8, 16.9)	11.0 (5.0, 19.6)	−0.3 (−2.2, 1.7)	0.78
Crisps and savoury snacks	8.1 (6.3, 13.8)	12.5 (6.3, 18.8)	9.0 (6.3, 17.3)	3.0 (0.9, 5.1)	*< 0.01*
Oily fish	37.5 (25.0, 53.0)	28.1 (16.5, 40.0)	32.3 (22.3, 50.5)	−4.2 (−9.7, 1.4)	0.14
White fish breaded or fried	30.0 (25.0, 50.0)	28.0 (25.0, 37.5)	30.0 (25.0, 45.0)	−6.2 (−12.3, −0.1)	*0.05*
Other fish dishes (incl. White fish and shellfish)	30.0 (18.0, 49.0)	28.8 (15.0, 43.3)	30.0 (16.3, 45.0)	−3.9 (− 9.4, 1.5)	0.16
Bacon and ham	25.0 (15.0, 48.8)	23.8 (11.3, 31.3)	23.8 (11.3, 32.5)	1.4 (−13.7, 16.5)	0.85
Pork and dishes	30.0 (22.5, 45.0)	30.0 (21.3, 42.8)	30.0 (22.3, 42.8)	1.6 (−3.7, 6.9)	0.55
Sausages	28.4 (15.0, 38.0)	22.5 (15.0, 39.9)	26.2 (15.0, 39.9)	−3.6 (−9.2, 2.0)	0.21
Beef, veal and dishes	34.7 (23.4, 53.3)	29.2 (17.5, 39.8)	32.0 (21.0, 50.0)	−4.1 (−10.4, 2.1)	0.19
Lamb and dishes	40.0 (25.9, 54.2)	23.5 (19.3, 36.5)	35.0 (22.5, 47.9)	−15.5 (−26.8, −4.3)	*< 0.01*
Liver and dishes	20.0 (10.0, 33.2)	20.0 (13.8, 25.6)	20.0 (12.5, 30.0)	1.2 (−5.1, 7.6)	0.70
Chicken and turkey dishes	56.2 (32.5, 83.4)	40.5 (26.3, 65.0)	48.8 (30.0, 77.5)	−11.3 (−18.3, −4.3)	*< 0.01*
Coated chicken	100.0 (60.0, 195.0)	95.0 (45.0, 125.0)	95.0 (45.0, 130.0)	6.0 (−51.4, 63.3)	0.84
Burgers and kebabs	25.0 (15.0, 53.8)	37.5 (8.5, 46.9)	25.0 (10.0, 50.0)	3.6 (−9.5, 16.7)	0.58
Meat pies and pastries	30.0 (22.5, 50.0)	37.5 (30.0, 45.8)	35.0 (25.0, 45.8)	0.5 (−9.4, 10.3)	0.92
Other meat and meat products	12.5 (6.3, 25.8)	12.5 (5.8, 24.8)	12.5 (6.3, 25.0)	−1.3 (−3.7, 1.2)	0.32
Chips, roasted and fried potatoes	46.0 (25.4, 64.4)	41.3 (25.0, 66.3)	41.3 (25.0, 64.4)	−1.4 (−6.8, 4.1)	0.63
Other potato dishes	60.0 (40.0, 93.3)	65.3 (42.5, 110.0)	62.5 (40.5, 101.8)	10.3 (2.6, 18.1)	*< 0.01*
Artificial sweeteners	1.0 (0.5, 2.6)	3.0 (0.9, 4.8)	1.5 (0.5, 3.8)	0.8 (−0.4, 2.0)	0.18
Dietary supplements	1.5 (1.0, 3.0)	2.0 (1.0, 3.0)	1.5 (1.0, 3.0)	−0.8 (−2.3, 0.6)	0.25
Tea, coffee and water	1288.4 (952.7, 1625.5)	1248.9 (899.3, 1635.5)	1264.3 (930.5, 1630.8)	15.3 (−64.1, 94.7)	0.71
Soft drinks low calorie	150.0 (82.5, 300.0)	142.0 (75.0, 325.0)	142.0 (75.0, 300.0)	6.7 (−96.1, 109.4)	0.90
Soft drinks not low calorie	100.0 (62.5, 160.8)	85.0 (50.0, 187.5)	91.8 (50.0, 175.0)	−15.2 (−53.0, 22.5)	0.43
Beer, lager, cider and perry	165.0 (75.0, 322.9)	310.0 (141.8, 496.1)	225.0 (110.0, 425.3)	176.3 (85.1, 267.5)	*< 0.01*
Wine	102.1 (43.8, 170.0)	129.0 (62.5, 218.8)	118.8 (46.9, 187.5)	27.1 (2.4, 51.7)	*0.03*
Spirits and liqueurs	21.0 (6.5, 25.9)	22.5 (11.5, 28.8)	22.5 (8.6, 28.8)	1.4 (−4.7, 7.5)	0.65
Miscellaneous	35.8 (14.0, 77.3)	34.3 (13.4, 81.3)	34.8 (13.8, 78.8)	−1.0 (−8.3, 6.2)	0.78

^a^Adjusted for age, sex, ethnicity, children, education, income and occupation

Note: Italics indicate statistical significance (*p* < 0.05)

**Table 10:** Median DASH scores in complete sample and analytic subsample

Sample	Participants (n)	DASH score (median (IQR))
Complete NDNS adult sample	6364	24 (20,28)
Analytic subsample	1063	30 (29,32)
